# H9N2 avian influenza virus in Muscovy ducks: a nationwide epidemiological survey and vaccine efficacy assessment

**DOI:** 10.1186/s13567-026-01857-0

**Published:** 2026-10-16

**Authors:** Xinwei Cao, Wenyang Li, Zelin Liu, Keyu Feng, Xinheng Zhang, Qingmei Xie

**Affiliations:** 1 State Key Laboratory of Swine and Poultry Breeding Industry & Guangdong Laboratory for Lingnan Modern Agriculture, College of Animal Science, South China Agricultural University, Guangzhou, 510642 China; 2 Guangdong Provincial Key Lab of AgroAnimal Genomics and Molecular Breeding, College of Animal Science, South China Agricultural University, Guangzhou, 510642 China; 3 Guangdong Engineering Research Center for Vector Vaccine of Animal Virus, Guangzhou, 510642 China

**Keywords:** H9N2 subtype avian influenza virus, Muscovy duck, coinfection challenge model, vaccine protective efficacy, antigenic drift

## Abstract

Avian influenza viruses (AIVs) are recognized as persistent threats to poultry health and global food security. The H9N2 subtype is widely distributed in poultry. A nationwide epidemiological survey was conducted in South, North, Central, and Southwest China, including Muscovy duck farms and live poultry markets, from 2022 to 2024. A total of 1208 Muscovy duck serum samples, 1212 duck-derived oropharyngeal/cloacal swabs and lung/tracheal tissues, and 480 chicken-derived samples were collected. In addition, 31 H9N2 strains were isolated, 2 of which were duck derived and 29 of which were chicken derived. A novel coinfection challenge model combining ocularnasal inoculation of H9N2 with injection of *Escherichia coli* (*E. coli*) was established, which induced severe tracheal lesions that closely resembled naturally occurring field pathologies. The coinfection challenge model successfully replicated prevalent field scenarios of viral‒bacterial synergism, resulting in severe lung lesions and a significant increase in viral loads. The results demonstrated that inactivated and live-vector vaccines provided more than 80% protection, with significant reductions in viral shedding and clinical symptoms. This study underscores the urgent need for updated vaccines matching circulating strains and the establishment of a validated framework for evaluating poultry vaccines under real-world coinfection scenarios.

## Introduction

Avian influenza viruses (AIVs), members of the influenza A virus family, have a broad host range, including poultry, wild birds, and humans, and are widely distributed across Eurasia and North America [1, 2]. Waterfowl are the natural reservoir of AIVs [3]. AIVs are transmitted through multiple routes, including avian migration, respiratory droplets, and fecal matter [4]. AIVs are categorized into 18 hemagglutinin (HA) and 11 neuraminidase (NA) subtypes on the basis of the antigenic diversity of these two proteins [5]. Among these, certain H5 or H7 subtypes, including H5N6 and H7N9, have been shown to be highly pathogenic, whereas the remainder, including H9N2, are considered to have low pathogenicity [6]. Despite its relatively low pathogenicity, the H9N2 subtype has emerged as the most prevalent and destructive subtype in poultry worldwide since it was first isolated in China in 1992, surpassing H5N6 and H7N9 [7]. Its persistent circulation is attributed to three main factors. First, avian migration contributes significantly to its transmission, facilitating pathogen exchange among avian populations. Second, contact between poultry and wild birds is a critical factor in viral spread, as it enables pathogen exchange through direct contact or via contaminated surfaces. Third, antigenic drift, arising from continuous viral evolution, leads to immune escape, rendering conventional vaccine-based control measures ineffective [8–10].

H9N2 low-pathogenicity avian influenza virus (LPAIV) poses a significant threat to both the poultry industry and public health. This threat is attributed to its broad host adaptability, antigenic drift, and role as a “gene donor” for highly pathogenic viruses (e.g., H5N1 and H7N9) [11]. Vaccination is widely recognized as a critical strategy for disease prevention [12]. However, continuous viral mutation has led to the reduced efficacy of traditional vaccines. Currently, inactivated vaccines, which are safe and cost effective, dominate the market [13]. However, inactivated vaccines are vulnerable to the effects of viral antigenic drift. The development of live attenuated vaccines offers a novel approach to address this issue. Live attenuated vaccines induce broad mucosal, cellular, and humoral immunity by mimicking the replication of natural infections, thereby conferring cross-protection. Although live attenuated vaccines exhibit strong applicability and developmental potential, attention should be given to the possibility of virulence reversion during application [14]. Another strategy for developing vaccines that mimic natural infections to induce immunity involves vector vaccines, which utilize specific carriers to express key antigens. Vector vaccines utilize carriers, including duck enteritis virus (DEV) and Newcastle disease virus (NDV), to express key H9N2 antigens (e.g., HA), thereby mimicking natural infections [15, 16]. These vaccines have the potential to be developed into multivalent vaccines. Another vaccine strategy that targets key antigens and holds potential for multivalent development is nucleic acid vaccines, which operate via a distinct mechanism involving the direct use of DNA or mRNA. Nucleic acid vaccines have been shown to target key antigens, including HA, and can be easily modified into multivalent vaccines [17].

To address these gaps, the present study was designed as a stepwise, hypothesis-driven investigation linking epidemiological surveillance, mechanistic pathogenesis modeling, and vaccine evaluation. Nationwide surveillance revealed widespread exposure of Muscovy ducks (*Cairina moschata*) to H9N2 viruses, despite low virus isolation rates, suggesting that infection may often occur in subclinical or transient forms under field conditions. However, severe respiratory disease is frequently observed in commercial farms, implying that additional cofactors may contribute to disease progression.

Based on these epidemiological observations, we hypothesized that secondary bacterial infection may play a critical role in exacerbating disease severity. Therefore, a sequential H9N2–*E. coli* coinfection model was established to better simulate natural infection conditions. Using this biologically relevant challenge model, we subsequently evaluated the protective efficacy of different vaccination strategies. This integrated approach provides a continuous framework linking epidemiological findings to mechanistic understanding and translational vaccine assessment.

## Materials and methods

### Samples and strains

Samples were collected from 2022 to 2024, including 1208 Muscovy duck serum samples; 1212 Muscovy duck-derived swabs (oropharyngeal/cloacal) and tissues (lung, trachea); and 480 chicken-derived samples, which were obtained from farms and live poultry markets in South, North, Central, and Southwest China.

The viral strains isolated in this study included 2 Muscovy duck-derived H9N2 strains (DK/GD/YF1/2024) and 29 chicken-derived H9N2 strains. The *E. coli* strain KP-24 was preserved in the laboratory. The commercial inactivated vaccine was purchased from Zhaoqing Dahuanong Biological Products Co., Ltd., and the live vector vaccine DEV-H9N2 was stored in the laboratory.

### GenBank accession numbers

The HA gene sequences of Muscovy duck-derived H9N2 strains obtained in this study were deposited in GenBank under accession numbers: PQ637449.1.

### Reagents and equipment

The key reagents used included specific pathogen-free (SPF) chicken embryos (purchased from Xinxing Dahuanong Poultry Egg Co., Ltd.), viral RNA extraction kits (Magen), a PrimeScript One-Step RT‒PCR Kit (TaKaRa), H9 subtype standard reference antigen (Harbin Guosheng Biotechnology Co., Ltd), 1% chicken red blood cells (prepared in-house), and formaldehyde (Shanghai Sangon Biotech).

The main equipment used included polymerase chain reaction (PCR) thermocyclers (Thermo Fisher), 37 °C incubators (Thermo Fisher), high-speed centrifuges (Thermo Fisher), and gel imaging systems (Syngene).

### Virus isolation and identification

Swabs and tissue samples were homogenized in PBS containing antibiotics, freeze-thawed three times, and centrifuged at 12000 rpm for 5 min to collect the supernatants. The supernatants (0.2 mL) were inoculated into 9–11-day-old SPF chicken embryos and incubated at 37 °C for 72 h, after which the allantoic fluids were harvested. HA-positive allantoic fluids were subjected to three blind passages for purification.

HA and hemagglutination inhibition (HI) tests were performed to identify viral activity. For molecular identification, reverse transcription (RT)-PCR was performed to amplify the HA genes via specific primers (HA-F: 5′- CAAGATGGAAGTAGTATCACT-3′; HA-R: 5′- TTGCCAATTATATACAAATGT − 3′), and the resulting PCR products were sequenced by Shanghai Sangon Biotech.

### Phylogenetic analysis

Nucleotide sequences of the HA gene were assembled via DNAstar Lasergene 11. A phylogenetic tree was constructed via MEGA-X software with the maximum likelihood method (1000 bootstrap replicates). Key amino acid sites, including HA cleavage sites, glycosylation sites, and receptor-binding sites, were analyzed via BioEdit.

### Statistical methods

Statistical analyses were performed using GraphPad Prism 10.4.2. Differences between groups were analyzed using two-way ANOVA. A *P*-value < 0.05 was considered statistically significant.

### Duck source and antibody status

Muscovy ducks were obtained from DP Breeding Duck Farm. Prior to the experiment, their H9N2 AIV antibody titers were confirmed to be 3.3 log_2_ by HI assay.

### Challenge model establishment

Given the discrepancy between high seroprevalence and low isolation rates observed in Muscovy ducks, we hypothesized that secondary bacterial infection may play a critical role in clinical disease manifestation. Therefore, a coinfection challenge model was established to simulate field conditions.

The 50 21-day-old Muscovy ducks were divided into five groups (Table 1). Viral shedding and tissue viral loads were detected via RT-quantitative (q)PCR, and pathological changes were observed at 5 and 10 days post-infection (dpi).

**Table 1:** **H9N2 challenge model experimental groups**

Group	Challenge strain	Challenge dose	Challenge route	Quantity	Day-old
1	H9N2 strain	10^7^EID^50^	Ocularnasal inoculation	10	21
2	*E. coli*	10^1.5^LD_50_	Intramuscular injection	10	21
3	H9N2 strain and *E. coli* coinfection	10^7^EID^50^; 10^1.5^LD_50_	Ocularnasal inoculation and intramuscular injection	10	21
4	H9N2 strain followed by *E. coli* at 5 dpi	10^7^EID^50^; 10^1.5^LD_50_	Ocularnasal inoculation and intramuscular injection	10	21
5	PBS	/	Ocularnasal inoculation and intramuscular injection	10	21

### Vaccine preparation

The H9N2 strain DK/GD/YF1/2024 was inactivated with 0.3% formaldehyde at 37 °C for 24 h, as confirmed by HA-negative results in SPF embryos. The inactivated antigen was emulsified with a mineral oil adjuvant at a 2:3 (v/v) ratio via a homogenizer (10000 rpm, 15 min). The emulsion stability was verified via centrifugation (10000 rpm, 3 min, no stratification), and the sterility was confirmed via TSA agar culture.

### Immunization and efficacy evaluation

Based on the optimized sequential coinfection model, we next evaluated whether currently available vaccine strategies could confer protection under realistic pathogenic synergism conditions.

The 50 1-day-old Muscovy ducks were divided into five groups (Table 2). At 21 days of age, groups A–D were challenged with H9N2 (10^7^ EID_50_) via the ocularnasal route, followed by intramuscular injection of *E. coli* (10^1.5^ LD_50_) at 5 dpi. Protective efficacy was evaluated on the basis of HI antibody titers, viral shedding suppression rates, tissue viral loads, and histopathological changes.

**Table 2:** **H9N2 vaccine efficacy evaluation experimental grouping**

Group	Immunization dose	Immunization route	Quantity	Immunization day-old
Inactivated vaccine	0.2 mL	Neck subcutaneous injection	10	1,7
Commercial vaccine	0.2 mL	Neck subcutaneous injection	10	1,7
DEV-H9N2 vector vaccine	0.2 mL	neck subcutaneous injection	10	14
Challenge control	/	/	10	/
Blank control	/	/	10	/

## Results

### Epidemiological surveys

Serological surveys of 1208 Muscovy duck serum samples (2022–2024) from South, North, Central, and Southwest China revealed an overall H9 antibody seropositivity rate of 69.7% (95% CI 67.0–72.3%) (Table 3).

**Table 3:** **Serum antibody seropositivity rates at poultry farms across the country**

Province	Farm	Day-old	Positive/total	Seropositivity (%)	Average H9 titer (log_2_)	95% CI (%)
Guangxi	JZ	70~315	17/164	10.4%	3.1	6.2–16.1
Fujian	JL	77~350	50/170	29.4%	2.67	22.7–36.9
Guangdong	DP	84~343	349/351	99.4%	9.11	98.0–99.9
Anhui	HY	49~329	137/192	71.4%	5.78	64.4–77.6
Anhui	GS	33~61	153/158	96.8%	7.48	92.8–99.0
Zhejiang	CS	34~58	136/173	78.6%	5.62	71.7–84.5
Total	/	/	842/1208	69.7%	6.14	67.0–72.3

The seropositivity rate was highest at the Guangdong DP farm and Anhui GS farm, with the geometric mean titer (GMT) reaching 9.11 log_2_ in some flocks, indicating widespread circulation of H9N2 in Muscovy duck populations.

**Figure 1:**
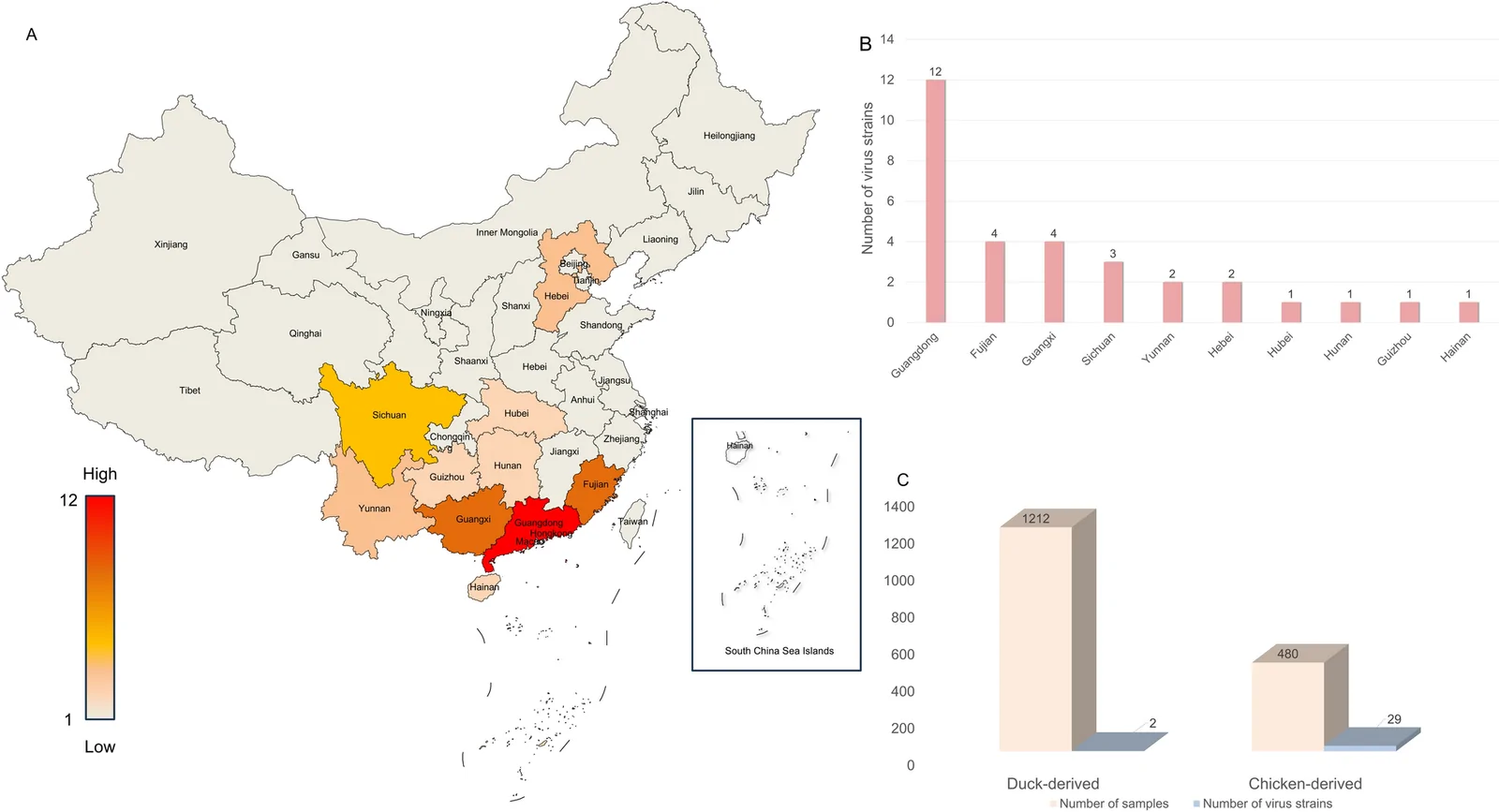
**Spatial distribution and host-specific statistics of H9N2 subtype avian influenza virus isolates in China (2022–2024).** **A** Geographic distribution of H9N2 isolates. The map shows the numbers of H9N2 virus strains and their distributions across various provinces/autonomous regions. **B** The number of H9N2 virus strains in each province. **C** The number of duck-derived and chicken-derived samples, as well as the number of isolated H9N2 virus strains.

H9N2 viruses were isolated from 2 of 1212 Muscovy duck samples (isolation rate: 0.2%, 95% CI 0.0–0.6%) and from 29 of 480 chicken samples (isolation rate: 6.0%, 95% CI 4.1–8.5%). These strains were distributed across Guangdong, Fujian, Hebei, Yunnan, and other provinces (Figure 1).

### Genetic characteristics of H9N2 isolates

**Figure 2:**
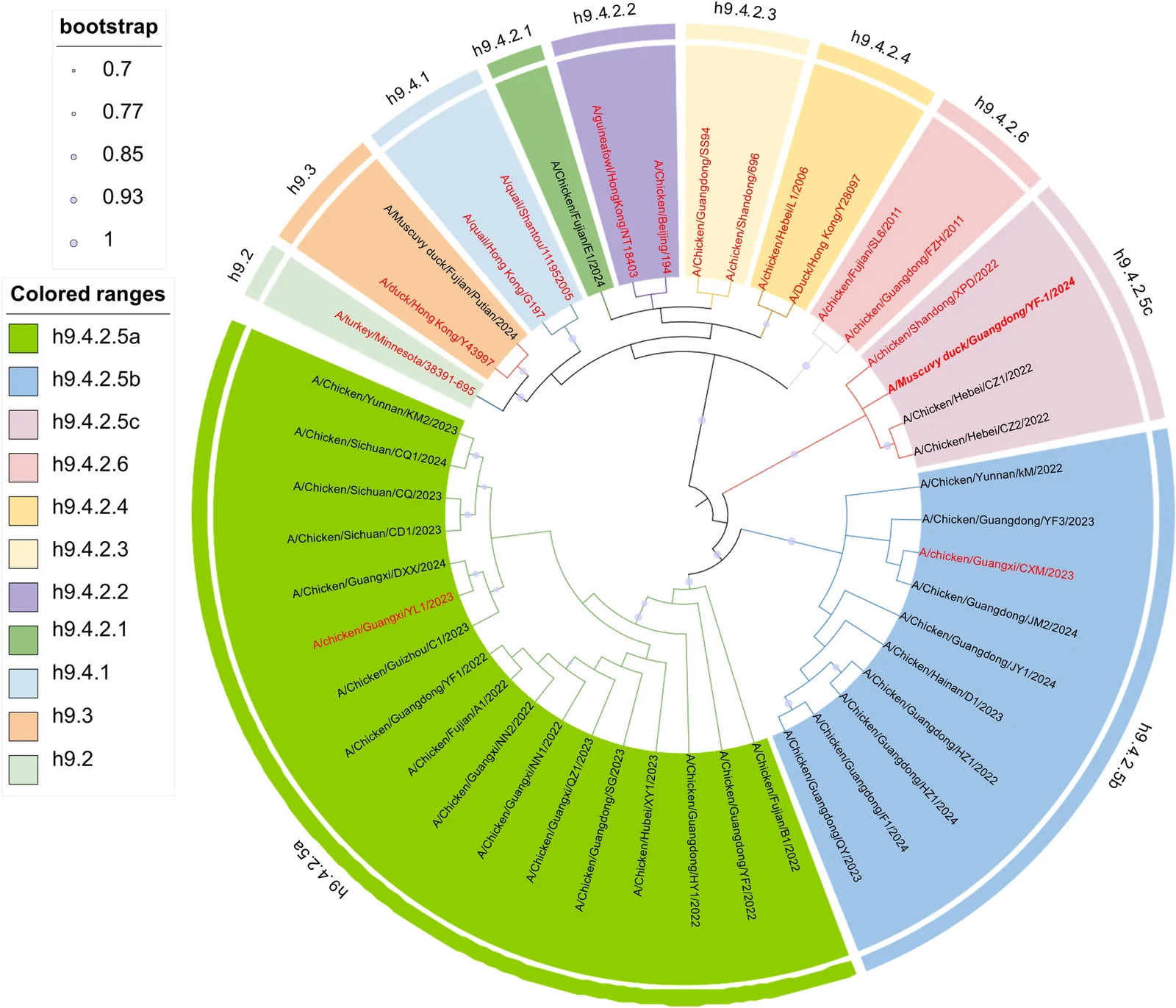
**HA gene phylogenetic tree.** Strains marked in red are reference strains; those marked in red, bold, and italics are the strains studied in this paper; all other strains were isolated between September 2022 and December 2024.

Phylogenetic analysis of the HA gene revealed that the 31 isolates clustered into four major lineages: H9.3, H9.4.2.5a, H9.4.2.5b, and H9.4.2.5c (Figure 2).

Most strains (19/31) belonged to clade H9.4.2.5a, whereas nine strains clustered within H9.4.2.5b, and three strains were grouped into H9.4.2.5c. Only one Muscovy duck-derived isolate (DK/FJ/PT/2024) belonged to clade H9.3, indicating the coexistence of multiple evolutionary branches in current circulation.

All isolates possessed low-pathogenic cleavage site motifs (PAGSSR↓GLF, PSRSSR↓GLF, or PAASDR↓GLF), lacking multiple consecutive basic amino acids, confirming their classification as LPAIVs. Notably, the PAASDR↓GLF motif was identified in two strains from distinct hosts, suggesting independent evolutionary events rather than clade-specific patterns (Table 4).

**Table 4:** **HA cleavage site analysis**

Strain name	Cleavage site location
A/Chicken/Sichuan/CQ1/2024	PAGSSR↓GLF
A/Chicken/Fujian/E1/2024	PSRSSR↓GLF
A/Chicken/Guangdong/HZ1/2024	PSRSSR↓GLF
A/Chicken/Guangdong/JM2/2024	PSRSSR↓GLF
A/Chicken/Guangdong/JY1/2024	PSRSSR↓GLF
A/Chicken/Guangdong/QY/2023	PSRSSR↓GLF
A/Chicken/Guangdong/SG/2023	PSRSSR↓GLF
A/Chicken/Guangdong/YF3/2023	PSRSSR↓GLF
A/Chicken/Guangxi/QZ1/2023	PSRSSR↓GLF
A/Chicken/Guangxi/YL1/2023	PSRSSR↓GLF
A/Chicken/Guizhou/C1/2023	PSRSSR↓GLF
A/Chicken/Hainan/D1/2023	PSRSSR↓GLF
A/Chicken/Hubei/XY1/2023	PSRSSR↓GLF
A/Chicken/Sichuan/CD1/2023	PSRSSR↓GLF
A/Chicken/Sichuan/CQ/2023	PSRSSR↓GLF
A/Chicken/Yunnan/KM2/2023	PSRSSR↓GLF
A/Chicken/Hebei/CZ2/2022	PSRSSR↓GLF
A/Chicken/Hebei/CZ1/2022	PSRSSR↓GLF
A/Chicken/Guangdong/HY1/2022	PSRSSR↓GLF
A/Chicken/Fujian/A1/2022	PSRSSR↓GLF
A/Chicken/Fujian/B1/2022	PSRSSR↓GLF
A/Chicken/Guangdong/YF2/2022	PSRSSR↓GLF
A/Chicken/Guangxi/NN1/2022	PSRSSR↓GLF
A/Chicken/Guangxi/NN2/2022	PSRSSR↓GLF
A/Chicken/Guangdong/HZ1/2022	PSRSSR↓GLF
A/Chicken/Guangdong/YF3/2023	PSRSSR↓GLF
A/Chicken/Yunnan/KM/2022	PSRSSR↓GLF
A/Muscovy duck/Fujian/Putian/2024	PAASDR↓GLF
A/Muscovy duck/Guangdong/yunfu-1/2024	PSRSSR↓GLF
A/Chicken/Guangxi/DXX/2024	PSRSSR↓GLF
A/Chicken/Fujian/E1/2024	PAASDR↓GLF

Analysis of receptor-binding site (RBS) sequences revealed four major amino acid patterns at positions 178–185, with substitutions observed primarily at positions 178 and 180. Although these variations were not strictly clade-specific, strains within clade H9.4.2.5a predominantly exhibited the PWNN*LY motif, whereas greater diversity was observed in H9.4.2.5c isolates. The left-hand side of the RBS pocket (positions 214–219) was relatively conserved (mainly NGLMGR), except for two strains presenting NGQQGR, indicating limited but detectable structural variation within the receptor-binding interface (Table 5).

**Table 5:** **HA gene RBS analysis**

Strain name	RBS	RBS left-hand side	RBS right-hand side
A/Chicken/Sichuan/CQ1/2024	PWTNALY	NGQQGR	GTSKA
A/Chicken/Fujian/E1/2024	PWNNVLY	NGLMGR	GTSNT
A/Chicken/Guangdong/HZ1/2024	PWTNTLY	NGLMGR	GTSTA
A/Chicken/Guangdong/JM2/2024	PWTNTLY	NGLMGR	GTSTA
A/Chicken/Guangdong/JY1/2024	PWTNTLY	NGLMGR	GTSTA
A/Chicken/Guangdong/QY/2023	PWTNTLY	NGLMGR	GTSTA
A/Chicken/Guangdong/SG/2023	PWNNVLY	NGLMGR	GTSNT
A/Chicken/Guangdong/YF3/2023	PWTNTLY	NGLMGR	GTSTA
A/Chicken/Guangxi/QZ1/2023	PWNNVLY	NGLMGR	GTSNT
A/Chicken/Guangxi/YL1/2023	PWNNVLY	NGLMGR	GTSNT
A/Chicken/Guizhou/C1/2023	PWNNVLY	NGLMGR	GTSNT
A/Chicken/Hainan/D1/2023	PWTNTLY	NGLMGR	GTSTA
A/Chicken/Hubei/XY1/2023	PWNNVLY	NGLMGR	GTSNT
A/Chicken/Sichuan/CD1/2023	PWNNVLY	NGLMGR	GTSNT
A/Chicken/Sichuan/CQ/2023	PWNNVLY	NGLMGR	GTSNT
A/Chicken/Yunnan/KM2/2023	PWNNVLY	NGLMGR	GTSNT
A/Chicken/Hebei/CZ2/2022	PWTNVLY	NGLMGR	GTSKA
A/Chicken/Hebei/CZ1/2022	PWTHALY	NGLMGR	GTSKA
A/Chicken/Guangdong/HY1/2022	PWNNVLY	NGLMGR	GTSNT
A/chicken/Fujian/A1/2022	PWNNVLY	NGLMGR	GTSNT
A/chicken/Fujian/B1/2022	PWYNTLY	NGLMGR	GTSNT
A/Chicken/Guangdong/YF2/2022	PWTNTLY	NGLMGR	GTSNT
A/Chicken/Guangxi/NN1/2022	PWNNVLY	NGLMGR	GTSNT
A/Chicken/Guangxi/NN2/2022	PWNNVLY	NGLMGR	GTSNT
A/Chicken/Guangdong/HZ1/2022	PWTNTLY	NGLMGR	GTSTA
A/Chicken/Guangdong/YF3/2023	PWNNVLY	NGLMGR	GTSNT
A/Chicken/Yunnan/KM/2022	PWNNTLY	NGLMGR	GTSNA
A/Muscovy/Duck/Fujian/Putian/2024	PWTHELY	NGQQGR	GTSKA
A/Muscovy/Duck/Guangdong/yunfu-1/2024	PWTNTLY	NGLMGR	GTSKA
A/Chicken/Guangxi/DXX/2024	PWNNVLY	NGLMGR	GTSNT
A/Chicken/Fujian/E1/2024	PWTHELY	NGQQGR	GTSKA

Potential N-glycosylation site analysis demonstrated two major glycosylation patterns associated with phylogenetic clustering. Strains in clades H9.4.2.5a and 5b typically possessed glycosylation sites at positions 29, 82, 141, 298, 305, and 313 in HA1, whereas strains belonging to clade H9.4.2.5c and some earlier isolates exhibited alternative glycosylation profiles (e.g., 11, 64, 123, 280, 287, 295). These clade-associated glycosylation patterns suggest evolutionary divergence in surface antigen configuration, potentially influencing antigenicity and immune recognition (Table 6).

**Table 6:** **HA gene potential glycosylation site analysis**

Strain name	Potential glycosylation site	
	HA1	HA2
A/Chicken/Sichuan/CQ1/2024	29 82 141 218 298 305	492 551
A/Chicken/Fujian/E1/2024	25 84 241 248 256	435 494
A/Chicken/Guangdong/HZ1/2024	29 82 141 298 305 313	492 551
A/Chicken/Guangdong/JM2/2024	29 82 141 298 305 313	492 551
A/Chicken/Guangdong/JY1/2024	29 82 141 298 305 313	492 551
A/Chicken/Guangdong/QY/2023	29 82 141 298 305 313	492 551
A/Chicken/Guangdong/SG/2023	29 82 141 298 305 313	492 551
A/Chicken/Guangdong/YF3/2023	29 82 141 298 305 313	492 551
A/Chicken/Guangxi/QZ1/2023	29 82 141 298 305 313	492 551
A/Chicken/Guangxi/YL1/2023	29 82 141 298 305 313	492 551
A/Chicken/Guizhou/C1/2023	29 82 141 298 305 313	492 551
A/Chicken/Hainan/D1/2023	29 82 141 298 305 313	492 551
A/Chicken/Hubei/XY1/2023	29 82 141 298 305 313	492 551
A/Chicken/Sichuan/CD1/2023	29 82 141 298 305 313	492 551
A/Chicken/Sichuan/CQ/2023	29 82 141 298 305 313	492 551
A/Chicken/Yunnan/KM2/2023	29 82 141 298 305 313	492 551
A/Chicken/Hebei/CZ2/2022	11 64 123 280 287 295	474 533
A/Chicken/Hebei/CZ1/2022	11 64 123 280 287 295	474 533
A/Chicken/Guangdong/HY1/2022	11 64 123 280 287 295	474 533
A/Chicken/Fujian/A1/2022	11 64 123 280 287 295	474 533
A/Chicken/Fujian/B1/2022	11 64 123 280 287 295	474 533
A/Chicken/Guangdong/YF2/2022	11 64 123 280 287 295	474 533
A/Chicken/Guangxi/NN1/2022	11 64 123 280 287 295	474 533
A/Chicken/Guangxi/NN2/2022	11 64 123 280 287 295	474 533
A/Chicken/Guangdong/HZ1/2022	11 64 123 280 287 295	474 533
A/Chicken/Guangdong/YF3/2023	11 64 123 280 287 295	474 533
A/Chicken/Yunnan/KM/2022	11 64 123 127 189 280 287 295	474 533
A/Muscovy/duck/Fujian/Putian/2024	11 64 123 200 280 287	474 533
A/Muscovy/duck/Guangdong/yunfu-1/2024	11 64 123 280 287 295	474 533
A/Chicken/Guangxi/DXX/2024	11 64 123 280 287 295	474 533
A/Chicken/Fujian/E1/2024	11 64 123 200 280 287	474 553

### Establishment of the H9N2–*E. coli* coinfection challenge model

The EID_50_ of the H9N2 strain DK/GD/YF1/2024 was 10^–6.5^/0.2 mL, and the LD_50_ of *E. coli* KP-24 in Muscovy ducks was 10^–5.5^ CFU/0.2 mL.

In the challenge experiment, no obvious clinical signs were observed in coinfected Muscovy ducks throughout the observation period at 1, 3, 5, 7, and 10 dpi. All ducks remained alert and maintained normal feeding behavior, with no detectable respiratory distress or neurological abnormalities.

Oropharyngeal and cloacal swabs were collected to assess viral shedding dynamics. Viral RNA was detected in 100% of ducks at 1 and 3 dpi. At 5 dpi, the positive rate decreased to 80%, followed by a marked reduction at later timepoints. By 10 dpi, only 20% of ducks remained positive, indicating progressive viral clearance over time.

**Figure 3:**
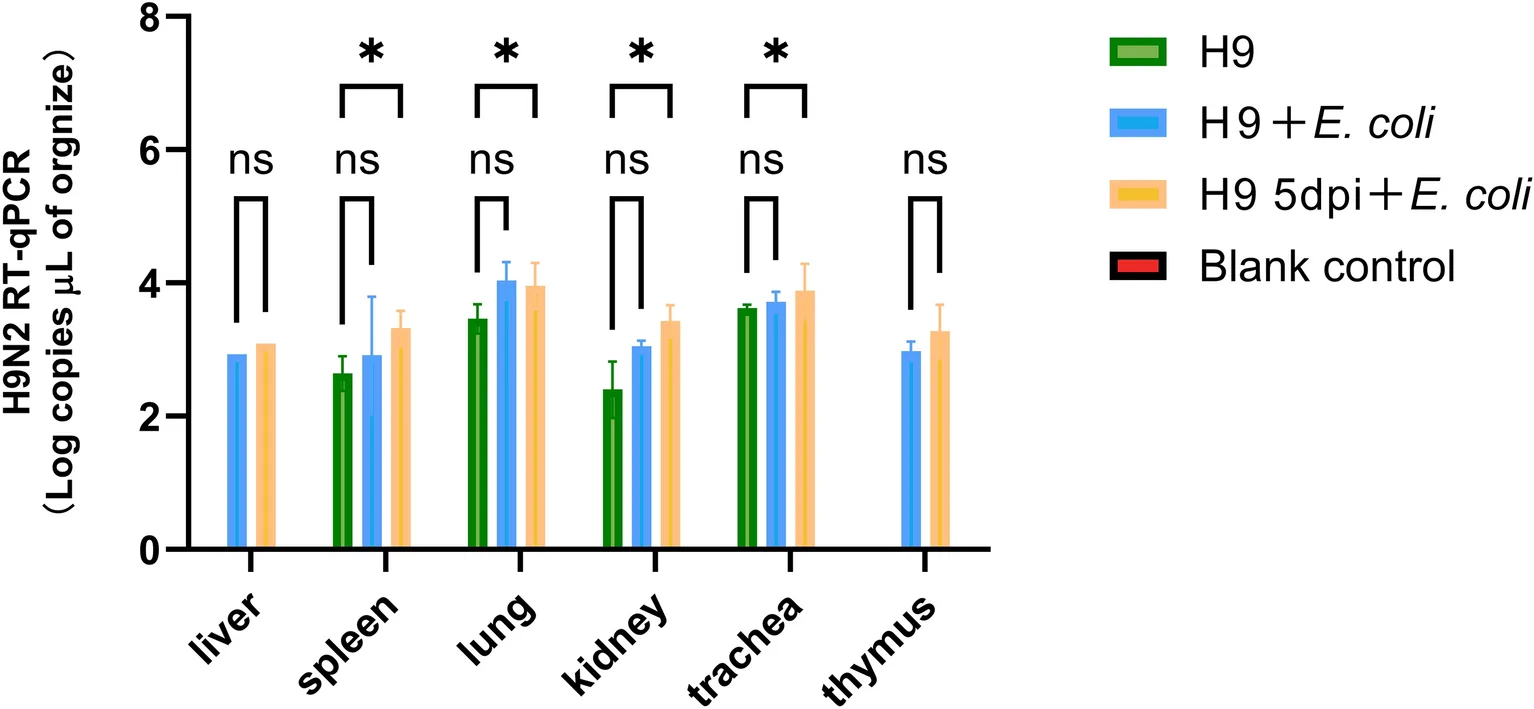
**Model tissue viral load**

Quantitative analysis of tissue viral loads showed that viral RNA was detectable in multiple tissues following infection. Notably, viral RNA was detected in the liver and thymus in the coinfection group but not in the H9N2 single infection group, suggesting that secondary *E. coli* infection may facilitate broader viral dissemination in vivo (Figure 3). No viral RNA was detected in the blank control group. These findings indicate that bacterial coinfection is associated with increased tissue distribution of the virus and may contribute to disease progression.

**Figure 4:**
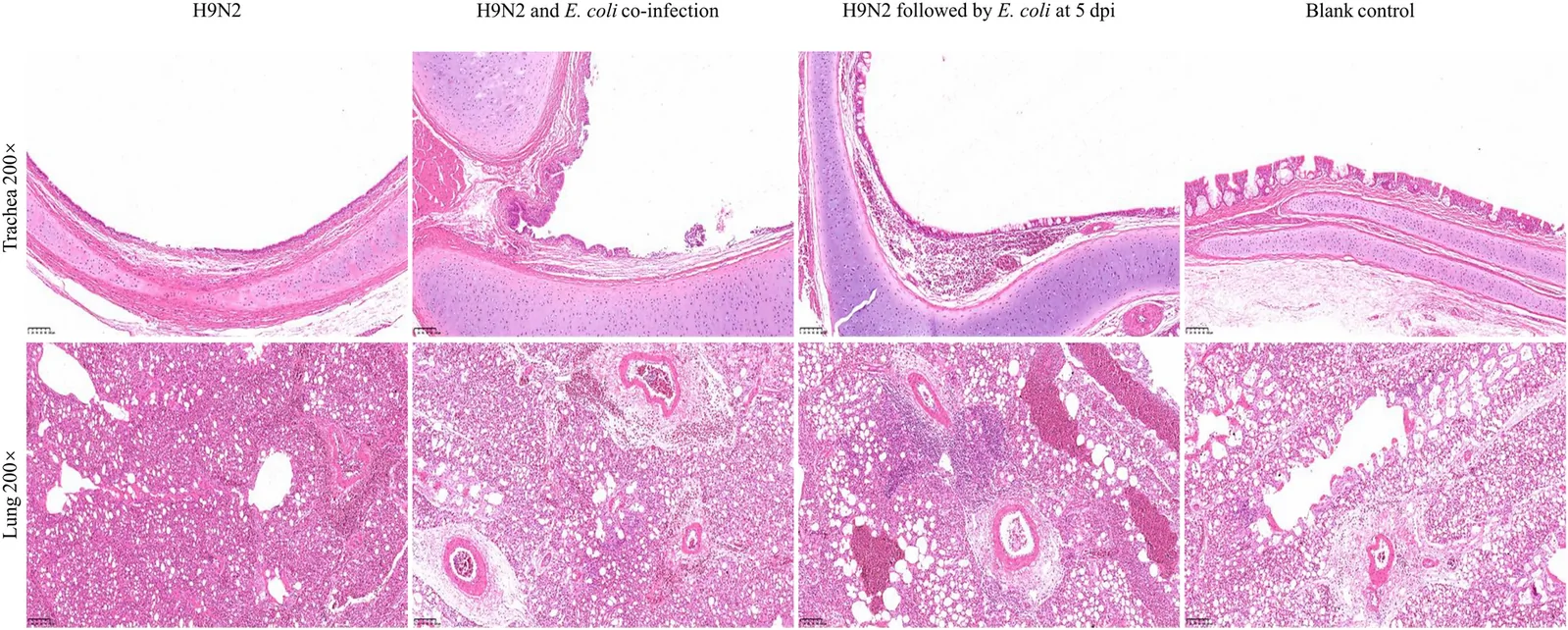
**Histopathological analysis of trachea and lungs in the H9N2 challenge model of Muscovy ducks at 5 dpi.** Histopathological analysis of the trachea at 5 dpi. H9N2 single infection group: reduced number of cilia with irregular arrangement and no other lesions; coinfection group: local mild submucosal epithelial detachment; sequential infection group: in addition to the above lesions, moderate submucosal edema and punctate inflammatory cell infiltration; blank control: normal structure. Histopathological analysis of the lung at 5 dpi. H9N2 single infection group: only mild damage; coinfection group: extensive moderate thickening of respiratory capillary walls and capillary congestion; sequential infection group: in addition to the above lesions, the presence of intrinsic lymphoid follicles, pervascular edema, hemorrhage, and more scattered inflammatory cell infiltration; blank control: normal structure.

Histopathological analysis confirmed tracheal mucosal damage and lung interstitial hemorrhage in this group, which is highly similar to natural infection (Figure 4).

### Vaccine efficacy evaluation

**Figure 5:**
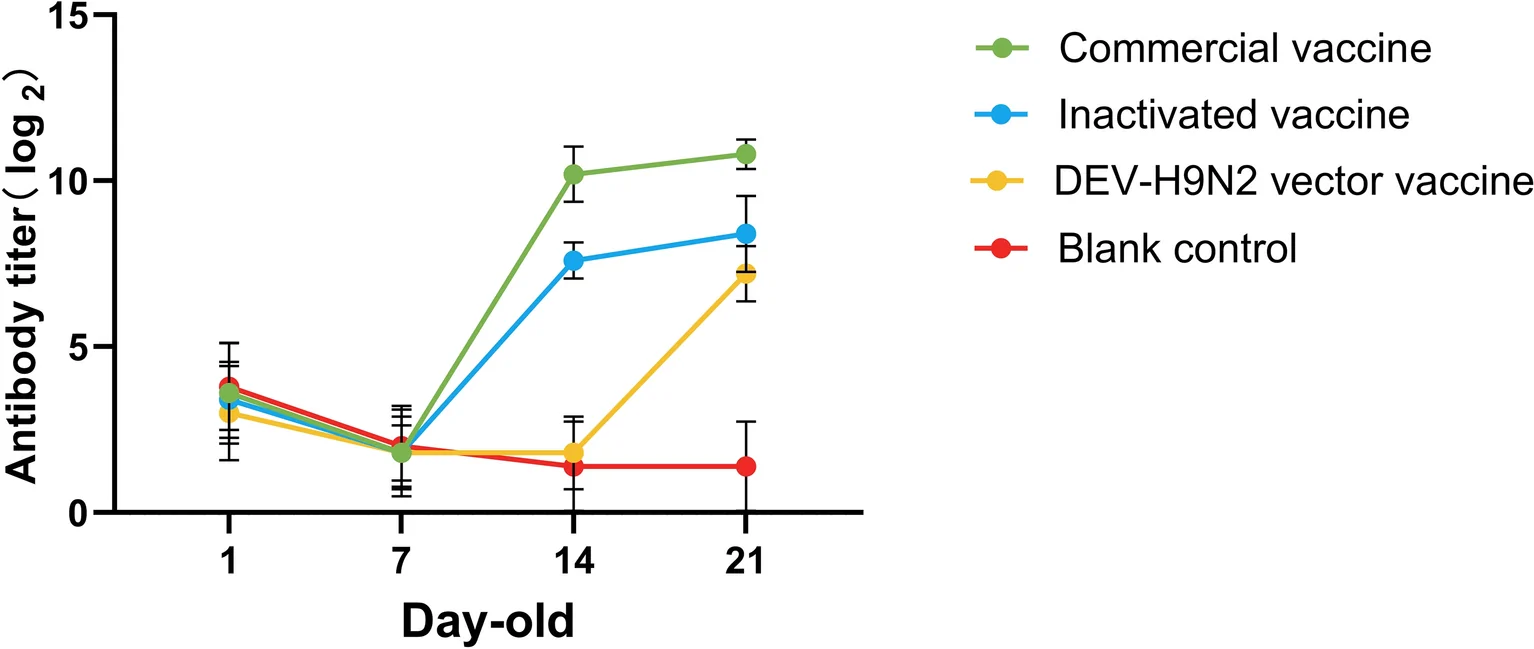
**Antibody levels in each immunization group**

Following immunization, the mean HI antibody titers were 8.4 log_2_ (inactivated vaccine), 10.8 log_2_ (commercial vaccine), and 7.2 log_2_ (DEV-H9N2 vector vaccine) (Figure 5).

Following H9N2 virus challenge, Muscovy ducks in the challenge control group exhibited typical clinical signs, including depression, ruffled feathers, and respiratory distress. In contrast, immunized ducks showed no obvious clinical abnormalities throughout the observation period.

Viral shedding was evaluated using oropharyngeal and cloacal swabs collected at 1, 3, 5, and 10 challenge post-inoculation (cpi). In the challenge control group, viral shedding was detected at all timepoints, although the shedding level decreased by 10 cpi, with an average viral titer of 2.2 log_2_. In contrast, viral shedding was detected in immunized groups at 1, 3, and 5 cpi, with significantly lower viral titers compared with the challenge control group. By 10 cpi, all immunized groups tested negative for H9 virus.

**Figure 6:**
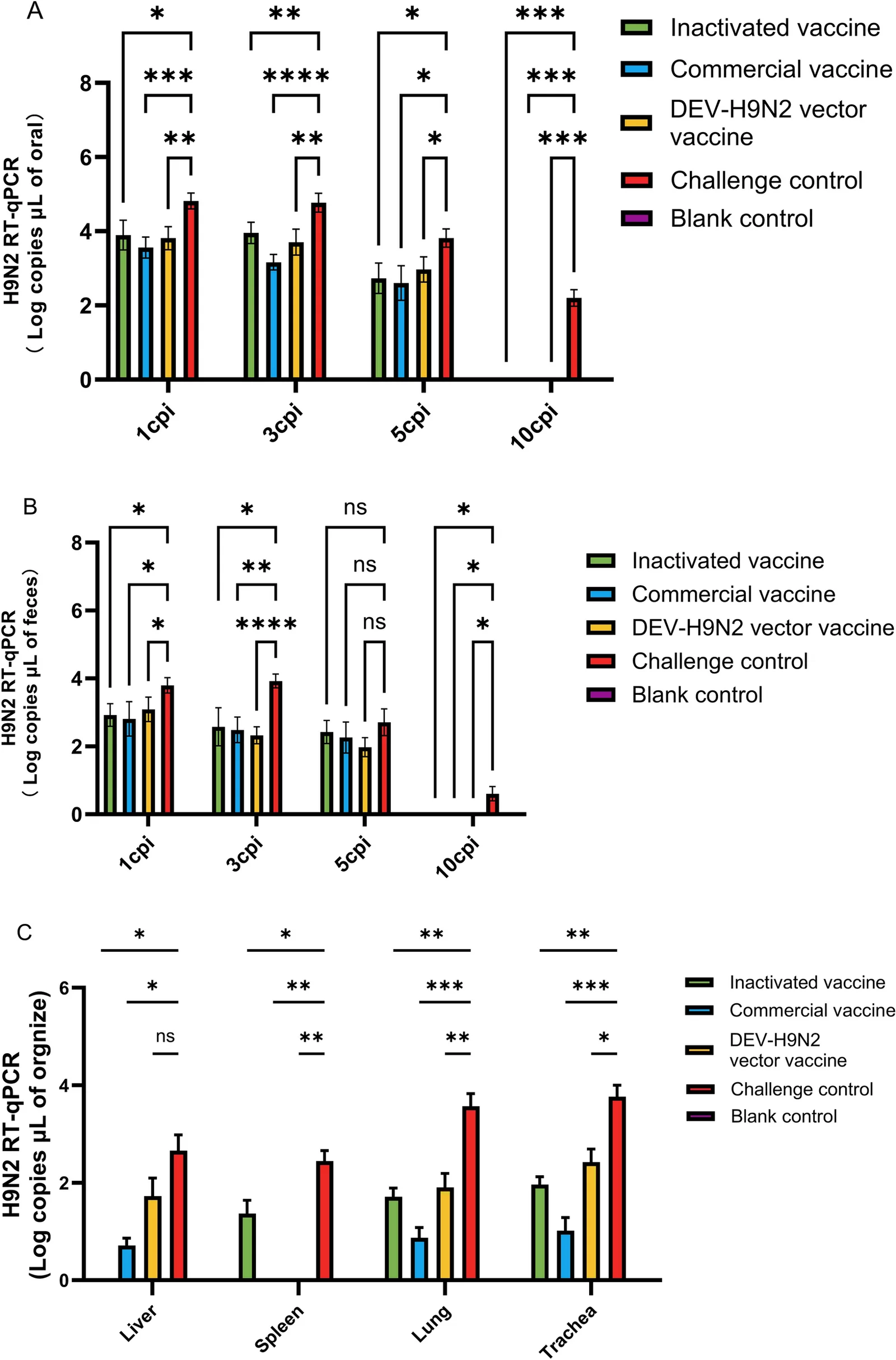
**Immunization protection-associated viral load.** **A** Oropharyngeal swab viral load. **B** Cloacal swab viral load. **C** Tissue viral load.

Additionally, viral load analysis in tissues demonstrated reduced viral replication in vaccinated ducks compared with the challenge control group, further confirming the protective efficacy of vaccination (Figure 6).

The viral shedding suppression rates at 3 cpi were 90% (inactivated vaccine), 90% (commercial vaccine), and 80% (DEV-H9N2 vector vaccine), with all groups exhibiting 90% suppression at 5 cpi and no shedding at 10 cpi (Table 7).

**Table 7:** **Swab-inoculated embryo hemagglutination results**

Group	3 cpi	5 cpi	10 cpi
Inactivated vaccine	1/10	1/10	0/7
Commercial vaccine	1/10	1/10	0/7
DEV-H9N2 vector vaccine	2/10	1/10	0/7
Challenge control	10/10	9/10	2/7
Blank control	0/10	0/10	0/7

**Figure 7:**
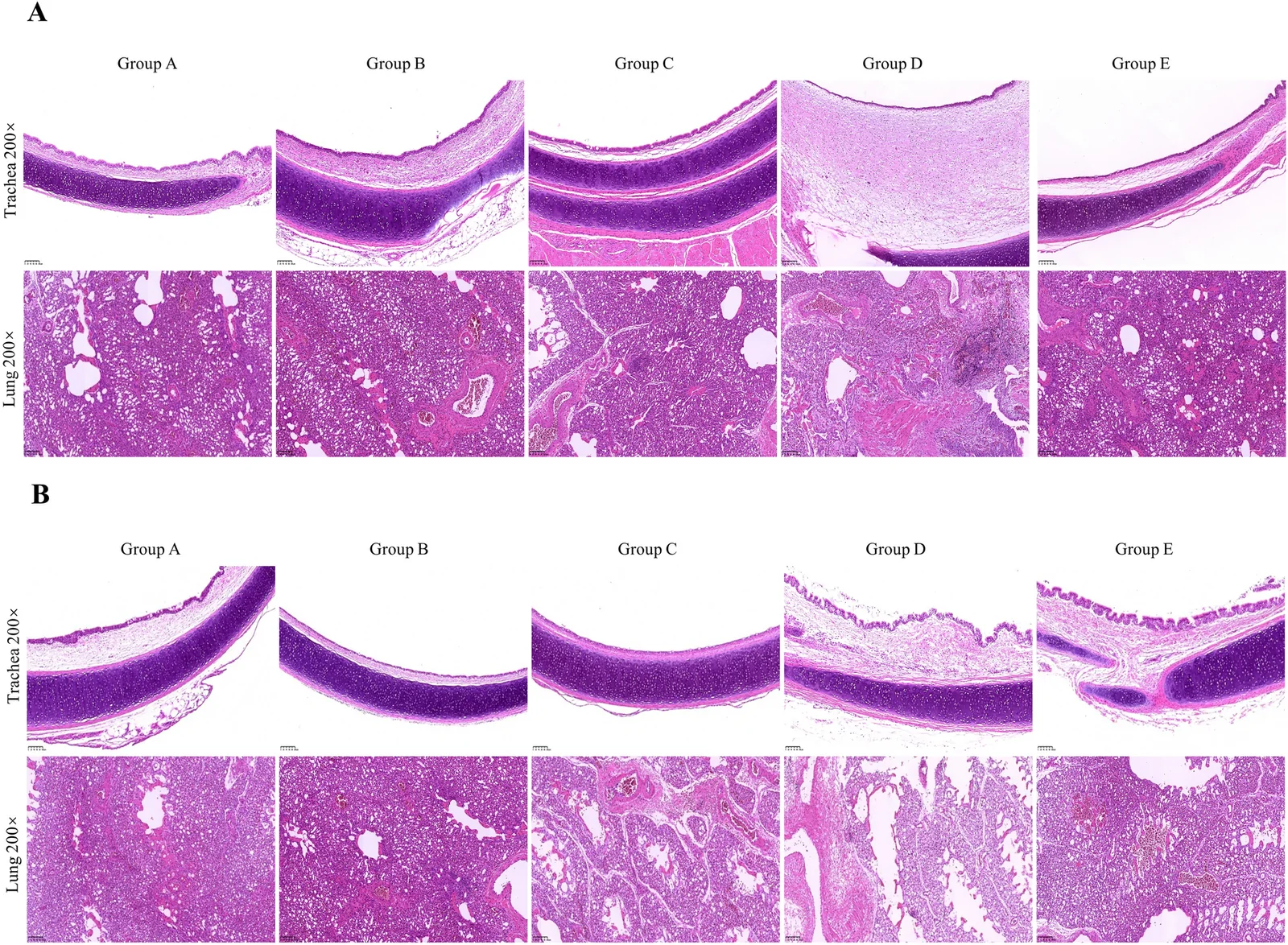
**Histopathological analysis of the trachea and lungs after challenge with H9N2.** **A** Histopathological analysis of the trachea and lung at 5 cpi. Control challenge: tracheal submucosal edema with massive inflammatory infiltration; lungs with extensive hemorrhage and inflammatory cells. Immune groups: patients with minimal or no lesions. Blank control: normal structure. **B** Histopathological analysis of the trachea and lung at 10 cpi. Challenge control: residual inflammation in the trachea and lungs. Immune groups: no obvious lesions were observed. Blank control: normal structure.

Pathology observations at 5 and 10 cpi revealed minimal lesions in the immunized groups, whereas the challenge control group presented severe lung congestion and tracheal inflammation (Figure 7). All three vaccines provided over 80% protection against the duck-derived H9N2 strain.

## Discussion

The H9N2 subtype, which is widespread in poultry populations worldwide, poses increasing cross-species transmission risks due to its expanding host range, which includes mammals [18–20]. Although the mortality rate of monoinfections rarely exceeds 20%, coinfections involving respiratory pathogens often result in severe disease and high mortality, inflicting substantial economic losses on the poultry industry [21–23]. Currently, inactivated vaccines are still employed as a primary control strategy against H9N2 outbreaks [24]. However, recent studies have reported that antigenic drift can impair vaccine efficacy [25]. To address these challenges, updated epidemiological data specific to understudied hosts, such as Muscovy ducks, are essential for refining control strategies.

Nationwide surveillance revealed a high seroprevalence of H9N2 antibodies in Muscovy ducks despite a relatively low virus isolation rate. This discrepancy suggests that H9N2 infection in this host species may often occur in subclinical or transient forms, resulting in widespread immune exposure without persistent viral detection [26]. Muscovy ducks occupy an important ecological position at the interface between aquatic and terrestrial poultry systems, and the detection of genetically diverse H9N2 viruses in this species indicates their potential role in viral maintenance and dissemination [27]. The widespread seropositivity observed in this study reflects extensive field exposure of Muscovy ducks to H9N2 virus. The use of a standardized H9 reference antigen in HI assays provides a consistent method for detecting HA specific functional antibodies across related H9 viruses. Although antigenic variation among different clades may influence detection sensitivity, HI assays remain the standard approach for assessing protective antibody responses relevant to vaccine evaluation and epidemiological surveillance [28].

Phylogenetic analysis showed that the isolates belonged to currently circulating poultry adapted H9N2 lineages [29]. Molecular characterization further revealed distinct receptor binding site sequence patterns and clade associated N glycosylation site configurations in the HA protein. Substitutions within the receptor binding region may influence receptor affinity and viral fitness, even when the overall receptor preference remains unchanged. Such sequence variability suggests ongoing adaptive evolution in Muscovy ducks and may contribute to antigenic diversification [30].

The identification of two predominant glycosylation patterns that correlate with phylogenetic clustering indicates structured antigenic remodeling of the HA surface. Glycosylation of the HA globular head is a well-recognized mechanism of antigenic drift [31]. The addition or relocation of glycans can reduce antibody accessibility to neutralizing epitopes while preserving viral functionality [32]. These structural modifications may facilitate immune evasion and allow viral persistence in populations with existing immunity. Such antigenic variation may gradually influence antigenic similarity between circulating strains and vaccine strains, thereby affecting long-term vaccine effectiveness [33–35].

H9N2 is classified as an LPAIV; however, it has been shown to weaken the immune system, disrupt the intestinal microbiota, and damage the intestinal mucosa [36–38]. In clinical cases, chickens infected with H9N2 exhibit severe respiratory symptoms, diarrhea, pericarditis, and other conditions [39, 40]. *E. coli* infection is considered the most prevalent underlying cause of these clinical signs [41, 42]. This suggests that secondary bacterial infections contribute significantly to disease severity. Based on this epidemiological context, a sequential H9N2 and *E. coli* coinfection model was established to better simulate natural infection conditions. Vaccine efficacy in this study was evaluated using this coinfection challenge model rather than a single virus challenge. Although the vaccines target H9N2 virus specifically, effective control of viral replication can reduce tissue damage and decrease susceptibility to secondary bacterial infection. The improved survival rates and reduced clinical severity observed in vaccinated groups under coinfection conditions indicate that vaccine induced antiviral immunity provides meaningful protection in complex infection scenarios.

Although obvious clinical symptoms were not observed in the coinfection model, viral shedding kinetics and tissue viral load analysis confirmed that viral replication occurred in infected ducks. The gradual reduction in viral shedding over time suggests progressive viral clearance. In this study, *E. coli* was included to increase model complexity and better simulate field conditions, where secondary bacterial infections commonly exacerbate influenza-associated disease [36, 41]. Consistent with this rationale, the coinfection model demonstrated enhanced viral dissemination and provided a more stringent and biologically relevant framework for evaluating vaccine efficacy under conditions that more closely resemble natural infections. Quantitative assessment of *E. coli* shedding was not performed, as the primary objective was to evaluate the impact of bacterial secondary infection on viral pathogenesis rather than bacterial transmission dynamics.

The observed reduction in clinical signs, viral shedding, and tissue viral load in vaccinated ducks confirms the protective efficacy of the vaccine. Importantly, vaccination accelerated viral clearance and prevented prolonged viral replication, which is critical for reducing transmission risk. These findings demonstrate that vaccination not only protects against clinical disease, but also limits viral dissemination, highlighting its value for controlling H9N2 virus in Muscovy duck populations.

Taken together, the integration of epidemiological surveillance, molecular analysis, coinfection modeling, and vaccine efficacy assessment provides a comprehensive framework for understanding H9N2 virus dynamics in Muscovy ducks. Continuous molecular and antigenic surveillance remains essential for monitoring viral evolution and optimizing vaccine strategies to ensure effective disease control.

## Data Availability

Data sharing is not applicable to this article as no datasets were generated or analyzed during the current study.
